# Kelps on the move: Potential future distribution areas in the face of climate change, on the Pacific coast of South America

**DOI:** 10.1371/journal.pone.0332591

**Published:** 2025-09-23

**Authors:** Milen Duarte, Natalia Sanhueza, Julio A. Vásquez, Fadia Tala, Alejandra V. González

**Affiliations:** 1 Instituto de Conservación, Biodiversidad y Territorio, Facultad de Ciencias Forestales y Recursos Naturales, Universidad Austral de Chile, Valdivia, Chile; 2 Instituto de Ecología y Biodiversidad, Barrio Universitario Concepción, Victoria, Chile; 3 Departamento de Ciencias Ecológicas, Universidad de Chile, Santiago, Chile; 4 Departamento de Biología Marina, Facultad de Ciencias del Mar, Universidad Católica del Norte, Coquimbo, Chile; 5 Centro de Investigación y Desarrollo Tecnológico en Algas y otros Recursos Biológicos-CIDTA, Coquimbo, Chile; 6 Instituto Milenio en Socio-Ecología Costera-SECOS, Coquimbo, Chile; MARE—Marine and Environmental Sciences Centre, PORTUGAL

## Abstract

Kelp forests are critical marine ecosystems that offer key services such as habitat, coastal protection, carbon sequestration, and support for fisheries. Along the temperate Pacific coast of South America, however, these seaweeds have historically been subjected to intense exploitation pressure, given their value as an economic resource. Additionally, they are impacted by oceanographic and climatic factors such as ENSO (El Niño-Southern Oscillation) event and ongoing climate change. The combined effects of these stressors pose a significant threat to their biomass and geographic distribution. Species distribution models under four representative concentration pathways for 2050 were used to assess the current and future potential distribution of two endemic intertidal kelp species, *Lessonia berteroana* and *Lessonia spicata*, which represent two of the four primary kelp species targeted by regional fisheries in the region. This approximation allows us to identify future areas of persistence (retained), retreat (lost), and expansion (gained). The results show that the environmental variables that mainly affected the potential distribution were salinity in *L. berteroana* and surface water temperature for *L. spicata.* The predictive models suggest that for *L. berteroana,* the lost area could reach 60.6%, and retained areas could account for 31.6% of the current area. Similarly, for *L. spicata*, the models indicate a potential loss of 58.6%, with retained areas comprising approximately 58.2% of the current area. Therefore, models predict a significant contraction could lead to the local disappearance of *Lessonia* species between 14° S and 25° S, profoundly altering coastal ecosystems and diminishing the critical ecosystem services they provide. Our modeling results underscore the urgent need for informed management and conservation strategies for kelp forests, which serve as vital ecosystem engineers. This research is especially critical in the face of climate change and ongoing anthropogenic pressures such as overexploitation. The study provides a robust scientific foundation for proactive measures to mitigate kelp forest decline and preserving their invaluable ecosystem functions along the Pacific coast of South America.

## Introduction

Anthropogenic factors and climate change affect marine biodiversity and species abundance in coastal areas [1,2]. These factors have the potential to significantly alter the distribution and diversity of marine life on a global scale [3]. For instance, species may undergo range reductions due to unfavorable conditions [4], survive only in isolated refugia where conditions remain suitable, or in some cases, expand their distributions into new areas [5]. These shifts can lead to substantial changes in biogeographic boundaries and increase the risk of local or global extinctions [6–8].

Shifts in environmental conditions that do not align with the niche requirements might lead to local or global extinction; conversely, species that are able to migrate can widen their distribution and shift their geographical boundaries [6]. The use of species distribution predictive models (Species Distribution Models-SDM) serves as a useful tool to assess current and future distributions under different hypothetical conditions (i.e., climate change) [9,10]. By comparing current and future model outputs, it is possible to identify which areas might maintain optimal climatic niche requirements, which can be classified as retained areas, as well as potential areas where local extinction might occur as lost areas, and areas where the species will expand, classified as gained areas [11,12].

Worldwide, kelps play a crucial role in marine ecosystems, contributing to primary productivity, biodiversity, and serving as structuring species for benthic communities, among other valuable ecosystem [13,14] and economic services [15]. These kelps thrive in intertidal and subtidal zones, and their distribution, growth, and reproductive traits are heavily influenced by abiotic factors [16]. Differential environmental thresholds are critical for each life-history stages, especially in microscopic gametophytes and juvenile stages [17–19]. This condition is not stable over space and/or time, especially in the context of climate change, and kelps, being one of the most abundant and extensively distributed seaweed species, are under critical danger [1,2,20].

Kelps of the genera *Lessonia* are one of the most conspicuous seaweeds, widely distributed along the coasts of South America, New Zealand, Tasmania, and Sub-Antarctic islands [21]. Along the Pacific coast of South America, six kelp species have been documented inhabiting the rocky shores between 15° and 56°S [21]. Among these, *Lessonia nigrescens* was historically considered a single species with a range spanning from 15° to 41°S, occurring in the lower intertidal zone in rocky areas exposed to waves. However, phylogenetic studies have revealed that what was once thought to be one species is actually composed of two closely related sibling species: *Lessonia berteroana* Montagne, distributed between 15° and 28°S, and *Lessonia spicata* (Suhr) Santelices, found between 31° and 41°S [22]. Both species provided a wide range of essential ecosystem services, including habitat provision, coastal protection, and supporting fisheries with economic values estimated at over USD 54 million [13]. However, these seaweeds have historically been subjected to intense exploitation pressure, especially in Northern Chile, due to their high alginate content, a natural polymer with medical and industrial applications, representing at least 70% of the national brown algae harvest [23]. In fact, Chile has positioned itself as the main exporter of kelp worldwide [24], with alginate extraction coming from natural populations. Like other kelps, *Lessonia* species offer multiple ecosystem services as habitat-forming species, which support intricate socio-ecological relationships with different actors in the fishery.

Both species are strongly influenced by oceanographic and climatic factors, including ENSO (El Niño-Southern Oscillation) events and the ongoing impacts of climate change. These combined stressors not only threaten to reduce their biomass but also to alter their geographic distribution. Given their disjointed ranges along the Pacific coast of South America and their critical role in the regional socioecological system, understanding potential future shifts in their distributions is essential for effective conservation and resource management. Such changes could have profound ecological and economic impacts, underscoring the urgency of proactive strategies.

This study aims to predict the current and future distribution of *L. berteroana* and *L. spicata*; we evaluated which areas may experience retention, loss, or gain by the climate model projection for 2040–2050, highlighting potential overlaps in distribution compared to near-current conditions. Both species are expected to shift their distributions toward higher latitudes, with the more equatorial species, *L. berteroana*, expected to be more affected than its congeneric counterpart species with a more southern distribution. Due to their economic and social value, these predictions are crucial for future planning and management of kelp forests in the Pacific coast of South America, providing insights information into intertidal habitat and complementing existing data on subtidal kelp forests regarding the giant kelp *Macrocystis pyrifera* [12].

## Materials and methods

### Study area and species

**Fig 1:**
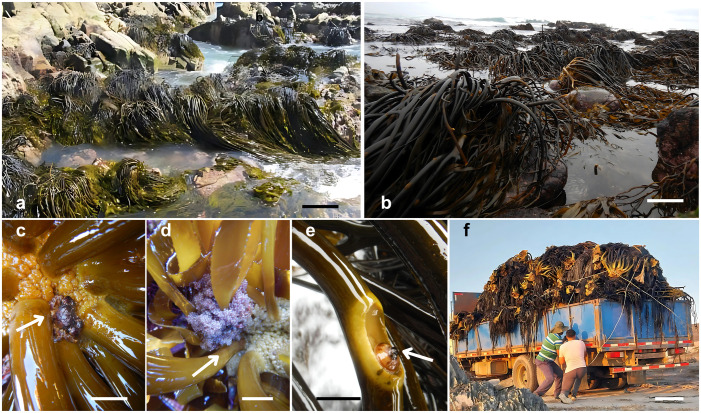
Field images of *Lessonia* species collected from Chilean TURF (Territorial Use Rights for Fishing) areas. **a)** *Lessonia berteroana* Montagne from TURF Punta Frödden, northern Chile (26° S). Scale bar = 50 cm. **b)** *Lessonia spicata* (Suhr) Santelices from TURF Chigualoco, central Chile (31° S). Scale bar = 30 cm. c–d) Close-up views of *L. berteroana* fronds densely colonized by a diverse assemblage of marine fauna, highlighting its role as a key ecosystem engineer. In **c)**, arrows indicate the crustacean *Acanthocyclus* sp., while in **d)**, arrows point to egg clutches of the intertidal fish *Myxodes* sp. Scale bar = 2.5 cm. **e)** Detail of a specialized herbivore-kelp association, with an arrow marking the limpet *Scurria* sp. Scale bar = 2.5 cm. **f)** Fishermen and a middleman’s truck loaded with harvested kelp highlighting the interactions between diverse actors in the kelp value chain. Scale bar = 50 cm.

The study area encompassed the current distribution of *L. berteroana* Montagne and *L. spicata* (Suhr) Santelices (Fig 1), extending from Marcona, Peru (15° S) to Chiloe (41° S) [22,25] with an isolated record located in southern Chile (48° S) [26]. Both species exhibit an overlapping distribution range between 28° S to 31° S. The habitat of both species consists of rocky substrates that are either exposed or semi-exposed to wave action within the low intertidal zone, where they create continuous belts of kelp forest providing essential resources for various marine organisms. Notably, *L. spicata* and *L. berteroana,* were previously described as *L. nigrescens* [22], underscoring significant taxonomic distinctions within this similar ecologically important kelp community. Historical data (references since 1950) were used, considering the distributional range of 15° S to 28° S to *L. berteroana*, and from 31° S to 41° S to *L. spicata*. Data collected as *L. nigrescens* within the overlapping area between 28° S–31° S, as well as the record of *L. spicata* [26] in southern Chile, were excluded from this analysis.

### Database

Species information was collected from the Global Biodiversity Information Facility [27], Google Scholar [28] (restricted to only public funded projects in Chile and Perú), and the ISI-Web of Science database [29]. A total of 242 publications were found for *L. berteroana* and 487 for *L. spicata.* Following the filtering of occurrence data to include records collected after 1950 with georeferenced information (latitude, longitude, DATUM), as well as detailed locality descriptions, 44 citations were selected (S1 Table). Additionally, georeferenced data were obtained from the Servicio Nacional de Pesca y Acuicultura of Chile [30] database, specifically from monitoring stations of the species of interest (S2 Table).

Once the spatially explicit database was constructed, the data were processed using QGIS [31] and Google Earth [32] to assess the spatial correspondence between the georeferenced data and the selected environmental layers for developing species distribution models (described below). This review led to the spatial correction of 30.95% of the data through the longitudinal linear adjustment from the coastline to the closest pixel of the environmental layers while maintaining the coordinate of the original data.

Current and future environmental variables were obtained from the Bio-ORACLE v2.0 [33,34], covering the RCP2.6, RCP4.5 and RCP6.0 and RCP8.5 scenarios for the 2000−2014- and 2040−2050 time frames [34]. The analysis focused on three available environmental variables: Water surface Temperature (°C) (maximum, mean, and minimum values); Salinity (PSS) (maximum, mean, and minimum values); and velocity (ms^-1^) (maximum and mean values).

### Species distribution models

To infer the potential distribution of *L. berteroana* and *L. spicata*, we constructed species distribution models [35] at a spatial resolution of five arcminutes, using MaxEnt v. 3.3.3 [36] with default settings, i.e., convergence threshold of 10^−5^, 500 iterations and a regularization multiplier of one, and 10,000 background points randomly selected from the model area because the fit is better for large areas [37]. Additionally, we randomly selected 25% of the total data as test data. The precision of each model was evaluated using the ROC curves and the AUC areas calculated by MaxEnt. Localities used for model calibration were excluded from the prediction. The logistic values obtained for each pixel were converted into presence/absence values using a threshold of 10 percentile training presence [38,39]; probability values below this threshold were classified as absent, while higher probabilities were classified as presence.

Separate models were run for each species and each RCP (2.6, 4.5, 6.0, and 8.5), resulting in four models for each species, encompassing both current and future versions. Following this, each model was adjusted to the current bathymetric occupancy using the Multibeam Bathymetry Database (MBBDB) from the National Center for Environmental Information of the National Oceanic and Atmospheric Administration-NOAA [40]. A depth threshold value was calculated for each *Lessonia* species according to current occurrence data and considering the intertidal habitat of the species, using the 10th percentile of occurrence data to define this threshold. The threshold value was then used to extract binary layers from the current and future models, resulting in a new refined model.

#### Retained, lost, and gained areas.

The latitudinal limits for the future potential distribution were calculated as the mean of the limits derived from the four resulting models. We overlapped current vs. future projections (ensemble approach; [39]). For each species and RCP scenario, three potential results were obtained: Retained (persistent areas where the current distribution coincides with the future projection), Lost (withdrawal zone, current distribution zones that are not occupied in the projection) and Gained (expansion area that is not currently occupied by the species). All spatial analyses were conducted using QGIS [31] and SDM Toolbox 1.1.c.

## Results

### Database

The data search returned in 1,488 occurrences, of which 336 (22.6%) were included in the species distribution models, with 200 corresponding to *L. spicata* and 136 to *L. berteroana* (S2 Table). Database curatorship excluded spatially duplicated records, data out of coastal limit, poor-georeferenced observations (e.g., geo-referenced observations in other latitudes), and records registered as *Lessonia nigrescens* in the overlap zone between *L. berteroana* and *L. spicata*.

### Species distribution

The results showed that all the models had considerably high accuracy, with a high AUC value averaging 0.995 for *L. berteroana* and 0.993 for *L. spicata*.

The principal environmental predictors of the potential distribution of *L. berteroana* were maximum surface salinity, minimum surface salinity, and mean surface temperature (Table 1). Maximum surface salinity contributed strongly (range in predicted high-suitability values 35.5–36.7%; Table 1). The current variable ranged from 27.74 to 35.84 PSS (S3 Table); within this range, *L. berteroana* showed a pronounced peak in occurrence or suitability centered at ~34.8 to 35.0 PSS, characterized by a strong, unimodal response curve, indicating a narrow optimum for this species (S4F Fig). Minimum surface salinity also had a strong effect (predicted high-suitability range 28.7–29.7%; Table 1). The current available range of this variable was 23.54–35.09 PSS (S3 Table) and the species showed a clear unimodal response, with suitability or occurrence peaking around ~34.5 PSS, indicating a relatively narrow salinity optimum (S4D Fig). Finally, mean surface temperature was an additional important predictor (predicted high-suitability range 18.4–20.6%; Table 1). The current available temperature range for this variable was 3.62–22.21 °C (S3 Table) and the specie response curve peaked between ~14.8 and ~17.5 °C and displayed a unimodal pattern (S4B Fig). These results indicate that *L. berteroana* shows highest predicted suitability at relatively high surface salinities and moderate surface temperatures.

**Table 1:** The Percentage contribution of environmental variables to the distribution models of *Lessonia berteroana* and *Lessonia spicata* for RCP2.6, RCP4.5, RCP6.0, and RCP8.5, calculated as percentage contribution. Sea surface temperature (°C) maximum, mean, and minimum; Salinity (PSS), maximum, mean, and minimum; and velocity (ms^-1^) maximum and mean. Major contributions are highlighted in grey.

Variable	Species							
	*L. berteroana*				*L. spicata*			
**RCP**	**2.6**	**4.5**	**6.0**	**8.5**	**2.6**	**4.5**	**6.0**	**8.5**
Maximum surface temperature	2.4	2.2	2.0	2.3	49.3	51.3	49.4	50.7
Mean surface temperature	18.5	18.4	20.6	19.8	17.0	17.4	17.0	16.6
Minimum surface temperature	0.1	0.1	0.1	0.1	2.9	2.8	3.0	3.6
Maximum surface salinity	35.9	36.7	35.7	35.5	1.5	2.5	1.3	1.9
Mean surface salinity	4.7	5.3	4.5	4.6	10.9	10.1	9.2	9.7
Minimum surface salinity	29.2	29.0	28.7	29.7	5.3	4.1	7.0	5.7
Maximum surface velocity	4.8	4.2	4.1	5.0	4.8	4.9	4.2	6.4
Mean surface velocity	4.4	4.1	4.2	2.9	8.2	6.9	8.9	5.4

In the case of *L. spicata*, the principal environmental predictors of the potential distribution were maximum surface temperature, mean surface temperature, and mean surface salinity (Table 1). Maximum surface temperature was the strongest contributor (contribution 49.3–51.3%; Table 1). The current total available temperature range of 5.70–27.10 °C (S3 Table) where the *L. spicata* species response in a unimodal response curve peaked mainly between ~14.8 and ~17.5 °C (S5C Fig). In the same way, the mean surface temperature also contributed substantially (16.6–17.4%; Table 1). The current available temperature range of 3.62–22.21°C (S3 Table) and the species response curve peaking between ~12.5 to ~14.8 °C and displayed a unimodal pattern (S5B Fig). Finally, the mean surface salinity was another predictor (9.2–10.9%; Table 1); with total available range between 25.39–35.40 PSS (S3 Table) The species salinity response curve was bimodal that peaked mainly between ~28.0 to ~33.6 PSS (S5E Fig), suggesting the presence of multiple salinity regimes that favor occurrence. Together, these results indicate that *L. spicata* has highest predicted suitability at intermediate to warm surface temperatures and moderate surface salinities.

**Fig 2:**
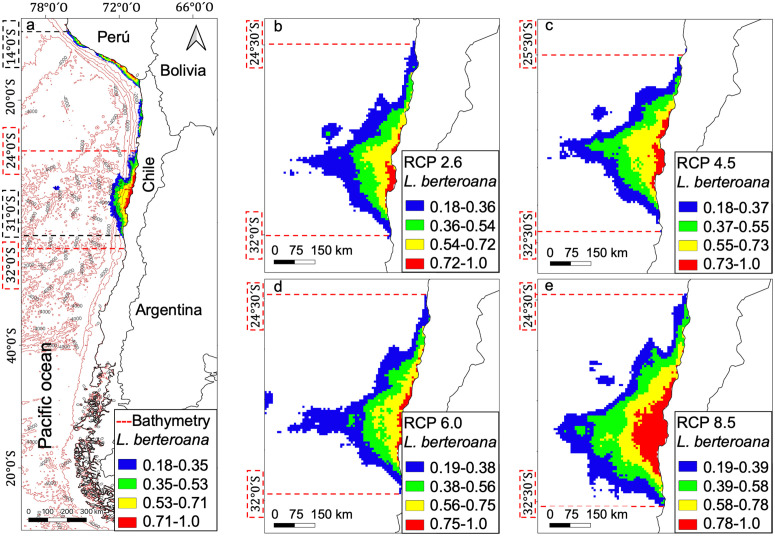
Species distribution models for *Lessonia berteroana,* current and future projections. **a)** Current distribution; b) future projections for the RCP2.6 scenario; **c)** RCP4.5 scenario; **d)** RCP6.0 scenario; and **e)** RCP8.5 scenario. Cold colors represent a lower probability, and warm colors indicate a higher probability of presence. Black dashed lines show the distribution limits (degrees), and red dashed lines denote the current position (degrees) of future projection limits.

**Fig 3:**
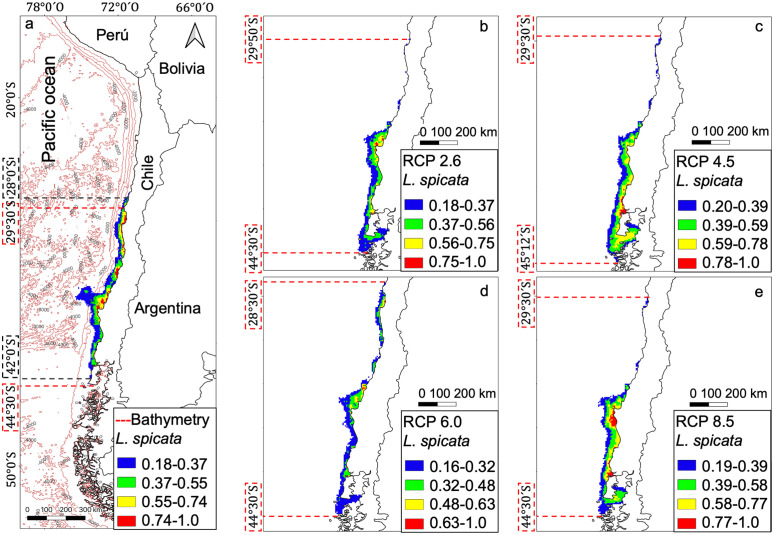
Species distribution models for *Lessonia spicata,* current and future projections. **a)** Current distribution; b) future projections for the RCP2.6 scenario; **c)** RCP4.5 scenario; **d)** RCP6.0 scenario; and **e)** RCP8.5 scenario. Cold colors represent a lower probability, and warm colors indicate a higher probability of presence. Black dashed lines show the distribution limits (degrees), and red dashed lines denote the current position (degrees) of future projection limits.

The current distribution model was developed with environmental variables and bathymetry for *L. berteroana* and *L. spicata*, obtaining a potential disjunct distribution. The models show that *L. berteroana* currently occupies a range between 14° S and 31° S (Fig 2A), while *L. spicata* occurs from 28° S to 42° S (Fig 3A). Future distributions models predict changes for both species, decreasing the northern limit for *L. berteroana* by an average of 10° (up to 24° S; Fig 2B–2E) and a reduction of one degree for *L. spicata* (up to 28° S; Fig 3B–3E). The southern limits are projected to increase by an average of one degree for *L. berteroana* (up to 32° S; Fig 2) and by two degrees for *L. spicata* (up to 44° S; Fig 3).

## Retained, lost, and gained areas

**Fig 4:**
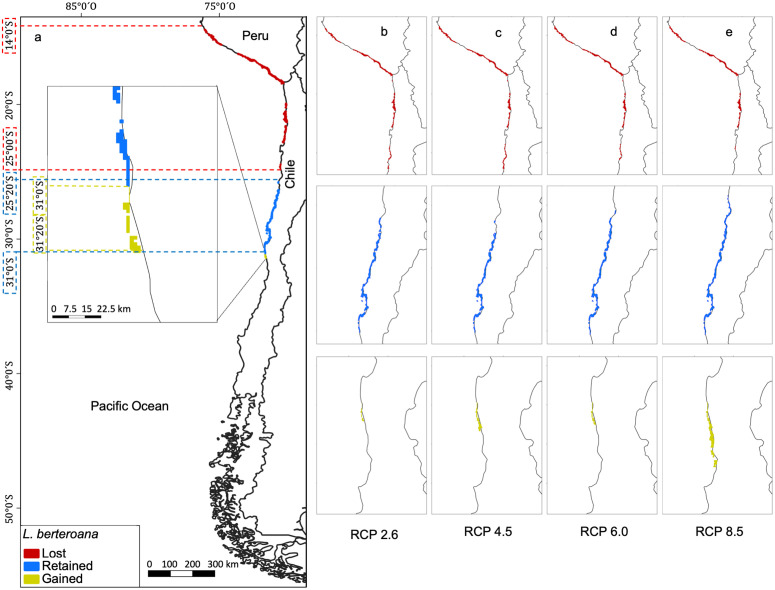
Retained, Lost, and Gained areas for *Lessonia berteroana.* Visualization of a) consensus model; b) future projections for the RCP2.6 scenario; **c)** RCP4.5 scenario; **d)** RCP6.0 scenario; and **e)** RCP8.5 scenario. Dashed lines indicated the position (degrees) of the distribution limits. Retained (blue), Lost (red), and potentially Gained (yellow) areas.

In the current model (Fig 4), *L. berteroana* occupies a total area of 876.8 km^2^ (Table 2). The overlap of both models, current and future distributions, indicates a loss of 531.2 km^2^ (Table 2), corresponding to the zone between 14° S and 25° S (Fig 4A). A retained area of 277.1 km^2^ (Table 2) corresponds to the zones between 25°20′ S to 31° S, while a gained area of 0.05 km^2^ corresponds to a small zone around 31° S (Table 2 and Fig 4A). Variation between RCP scenarios is minimal, except for RCP8.5 (Fig 4E), where the area of potential expansion could be 0.591 km^2^.

**Table 2:** Values for the consensus models for RCP2.6, RCP4.5, RCP6.0, and RCP8.5 scenarios, showing the percentage of area, current distribution area, retained area, lost area, and gained area in km^2^ for *L. berteroana* and *L. spicata.*

	Species							
	*L. berteroana*				*L. spicata*			
**Scenario**	**Current**	**Retained**	**Lost**	**Gained**	**Current**	**Retained**	**Lost**	**Gained**
RCP2.6	910.4	296.3	614.1	0.07	510.1	272.8	237.3	204.7
		(32.6%)	(67.5%)			(53.5%)	(46.5%)	
RCP4.5	926.0	277.0	648.9	0.02	537.8	270.3	267.5	553.5
		(29.9%)	(70.1%)			(50.3%)	(48.7%)	
RCP6.0	932.0	321.7	610.3	0.07	526.9	139.5	387.4	0
		(34.5%)	(64.7%)			(26.5%)	(73.5%)	
RCP8.5	924.7	337.8	586.9	0.59	546.2	276.4	269.8	347.9
		(36.5%)	(63.5%)			(50.6%)	(49.4%)	
Consensus	876.8	277.1	531.2	0.05	479.0	278.9	280.9	156.0
		(31.6%)	(60.6%)			(58.2%)	(58.6%)	

**Fig 5:**
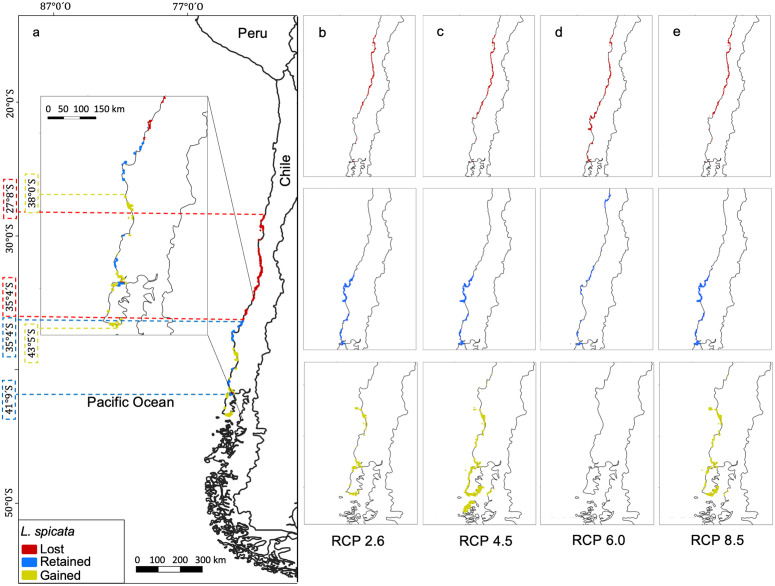
Retained, Lost, and Gained areas for *Lessonia spicata.* Visualization of a) consensus model; b) future projections for the RCP2.6 scenario; **c)** RCP4.5 scenario; **d)** RCP6.0 scenario; and **e)** RCP8.5 scenario. Dashed lines indicated the position (degrees) of the distribution limits. Retained (blue), Lost (red), and potentially Gained (yellow) areas.

In the current model, *L. spicata* occupies a total area of 479.0 km^2^ (Table 2). The superposition of the two models, current and future distributions, indicates a loss of 280.9 km^2^ (Table 2) corresponding to the zone between 27°8′ S and 35°40′ S (Fig 5A). A retained area of 278.9 km^2^ (Table 2) corresponds to the zones between 35°40′ S and 41°9′ S (Fig 5A), and a gained area of 156.0 km^2^ (Table 2) corresponds to the zone between 38° S and 43°5′ S (Fig 5A). Variation between RCP scenarios is minimal, except for RCP 6.0 (Fig 5D), where the gained area is 0 km^2^.

## Discussion

*Lessonia berteroana* and *L. spicata* form a continuous intertidal belt on rocky exposed coasts in Peruvian and Chilean shores, spanning from 14° and 42° S. Both species exhibit a small overlap in distribution around 30° S, where they experience similar environmental pressures despite being different species. This unique biogeographical setting makes them an ideal natural model for studying and predicting the potential impacts of climate change and other environmental drivers across both temporal and spatial scales.

Our integrative analysis using species distribution models (SDMs) that incorporate key environmental parameters temperature, salinity, and velocity has identified the principal factors shaping the potential distributions of each species*.* For *L. berteroana*, mean surface temperature and extreme fluctuations in surface salinity (both maximum and minimum) emerged as the strongest predictors delimiting its range. In contrast, the potential distribution of *L. spicata* is primarily influenced by maximum and mean surface temperature alongside mean surface salinity. These species-specific environmental sensitivities underscore distinct ecological niches and adaptive capacities between the two kelps. Although these results highlight the key parameters likely to influence kelp species distributions under climate-change scenarios, it is important to acknowledge that multiple environmental factors constrain both macroscopic and microscopic life stages across the study area [e.g., 19,21,41–44], complicating the interpretation and mechanisms that ultimately determine the distribution model outputs. Crucially, our model-derived inferences align closely with experimental evidence describing physiological constraints across different life-history stages of these species. At the regional scale, *L. berteroana* populations inhabiting northern latitudes experience a hydrological regime marked by reduced freshwater inputs from precipitation and glacial meltwater, resulting in a relatively narrow and stable salinity regime (~34.5 to ~35.0 PSS) with strength and unimodal response curve. By contrast, *L. spicata* is exposed to a much broader salinity gradient (~22.5 to ~ 34.5 PSS) and shows a wider, more variable response curve with indications of multiple peaks. At the local scale, both intertidal species undergo extreme low tide exposures, which produce pronounced fluctuations in desiccation, temperature, and salinity [e.g., references 18,41], further modulating their distribution and persistence.

Consistent with our modeling outcomes, experimental data demonstrate that temperature and salinity critically influence reproductive and developmental processes in kelps [17,42–45], governing the timing and success of reproductive onset [18,19]. Specifically, *L. berteroana* gametophytes exhibit greater tolerance to elevated temperatures, whereas *L. spicata* gametophytes are better adapted to cooler conditions [19], patterns that align directly with the observed latitudinal distribution of mature sporophytes. Furthermore, because *L. berteroana* occupies a narrow and stable salinity range, it could be more susceptible to projected increases in surface salinity under future climate scenarios (0.24–0.46 PSS for RCP 8.5), since these changes may exceed critical developmental thresholds. Similar vulnerabilities have been reported for *Macrocystis pyrifera*, which co-occurs with both *Lessonia* species, highlighting broader ecological risks associated with environmental fluctuations within coastal ecosystems [17].

Together, the strong concordance between our species distribution models and empirical physiological data validates our predictive framework and underscores urgent ecological vulnerabilities. These findings emphasize the imperative for targeted conservation and management strategies that explicitly consider species-specific environmental tolerances. Addressing these challenges in the face of increasingly dynamic coastal environments will be essential for preserving the resilience and functional integrity of these foundational kelp ecosystems.

According to the consensus model, predictive models indicate that the distribution for both species will vary drastically along the southeastern Pacific coast, with a projected reduction in size by more than 60% for *L. berteroana* and 58% for *L. spicata* relative to their current areas. However, some potential future refugia and areas for expansion could be colonized. The current range of *L. berteroana,* of nearly 17° (14°-31° S) could decrease by 11°, corresponding to approximately 1.221 km^2^ (60.62% of current), leaving a refuge area between 25° S to 31° S (~6°), and allowing for an expansion of just over one degree. The current range of *L. spicata*, about 14° (27° S to 41° S), is projected to be reduced by 7°, corresponding to approximately 777 km^2^ (58.6% of its current area), maintaining a refuge just between 35°S to 41° S (~6°) and potentially expanding another 5° into higher latitudes. The current small overlapping distribution around 30° S is expected to disappear according to these predictions.

Along the southeast Pacific coast, climate models predict that the increase in ocean surface temperature will be less intense towards higher latitudes [12,46], which could generate differential changes in the distribution of the species that inhabit the coastline. Our results, based on changes in temperature, salinity, and surface velocity, predict that the distribution of *L. berteroana* and *L. spicata* are expected to decline by at least 50% in the next 50 years, levels that are considered endangered according to the International Union for Conservation of Nature’s Red List [47]. Therefore, the results of the distribution models may be further affected if harvesting pressure increases, potentially reducing population abundance, suggesting that specific coastal areas are at significant risk and need the protection and conservation of kelps. Currently, the Chilean coastline lacks no-take marine protected areas that include these potential refuge areas for both species of *Lessonia*. Thus, these results could serve as valuable input for specific marine conservation planning.

The projected lost, retained, and gained areas for *Lessonia* spp. from low to high latitudes of our models align with global projection for giant subtidal kelp *Macrocystis pyrifera*, which coexists with both *Lessonia* species in these regions. This congruence highlights a decline in suitable habitat at lower latitudes and an expansion at higher latitudes [12]. Development biological strategies for adaptation to these changes in order to mitigate loss of habitat, requires significant knowledge and technological advancement [12,48,49]. This task is particularly complex due to the speed of environmental change. In this context, the models can be invaluable for managing species based on specific areas. While these predictions must be cautiously approached using systematic procedures for distribution modeling [50]. Under these scenarios, preventing the loss of these essential endemic *Lessonia* species will require effective management strategies, including conservation, restoration, and afforestation efforts [2]. Interventions aimed at mitigating the potentially significant consequences of decreased suitable habitat for *Lessonia spp*. are crucial for safeguarding natural kelp populations and their numerous benefits, such as the numerous benefits they provide, including marine biodiversity [51], carbon sequestration [52], and alginate commercialization [13]. These factors are all tied to a complex and vital socio-ecological system in the Southeast Pacific region.

The management of retained areas could be assessed as genetic refuges for seaweed species [53,54]. These habitats are essential not only for the survival and genetic diversity of foundational seaweeds but also for the broader marine communities that depend on them [55]. As ecosystem engineers, these seaweed species significantly shape their environments by creating complex physical structures that support high levels of biodiversity. Through their presence, they enhance habitat complexity, provide shelter and food resources, and facilitate critical ecological interactions across multiple trophic levels [56]. Maintaining such areas is therefore fundamental for preserving marine ecosystem resilience, productivity, and the intricate web of life they support.

To effectively preserve these kelp populations, strict conservation measures are crucial, not only to mitigate the escalating impacts of climate change but also to address other pressing threats such as overexploitation and anthropogenic perturbations arising from productive activities like coastal development and pollution, which pose significant risks to marine ecosystems. Despite the ecological significance of these natural refuges, there are currently no marine protected areas (MPAs) explicitly designated for their conservation. Nevertheless, between 28°S and 29°S, where the predicted refuge of *L. berteroana* is located, the Humboldt Archipelago Multi-Use Marine Coastal Protected Area (AMCP-MU) provides an existing framework for habitat protection. Implementing strict prohibitions on the extraction of seaweed from natural banks within such protected zones, especially in areas like the AMCP-MU, constitutes one of the most effective conservation strategies available. Moreover, Chilean legislation through the Territorial Use Rights for Fisheries (TURF) system offers a significant tool for sustainable management and conservation. TURF programs grant local fishing communities exclusive rights to harvest resources in specific areas, incentivizing long-term stewardship. Studies demonstrated that several TURF organizations operate sustainably, balancing ecological, social, and economic perspectives [57]. These programs have successfully implemented proactive conservation measures, including rotating harvest zones and establishing no-take reserves, to ensure continuous resource availability and enhance ecosystem resilience [58]. However, persistent regulatory noncompliance such as illegal activities and disregard for local TURF rules (pers. comm. R. Martínez, TURF Chigualoco) remains a critical challenge. Accelerating improvements in surveillance, increasing penalties, and strengthening both formal and informal co-management are urgently needed to ensure compliance [59]. Under this scenario, without prompt and decisive action in conservation, management, and enforcement, the protection of Chile’s kelp populations, their refuges, and the marine biodiversity they sustain faces an urgent and serious threat.

Natural colonization of *Lessonia* spp. species in the projected gained areas may face limitations such as geographical barriers, competition with other species, and low dispersal capacity [25,59]. In the overlapping distribution area in central Chile, some *Lessonia* species compete for space with species like *Durvillaea incurvata* (comb. nov. *D. antarctica*; [60]), which could restrict the expansion of *L. spicata*. Additionally, various intertidal factors, including the temporal changes of sandbanks, waves, tides, swells, and upwellings, affect the microscopic phase of *L. spicata*, impairing its ability to generate a seed bank, and reducing its capacity for dispersal and genetic flow beyond 4 km [21,60]. However, *L. spicata* could potentially expand its distribution to 43° S, with the possibility of expanding into Golfo of Penas [26]. This result suggests that, despite evidence of limitations due to competition and dispersal, environmental conditions may be conducive to increased colonization at higher latitudes, as emphasized in our models. Nonetheless, the ecological uncertainty associated with this potential increase in colonization underscores the need for appropriate monitoring to facilitate informed and timely decision-making.

Ultimately, the species distribution models for *L. spicata* and *L. berteroana* provide a crucial baseline for careful planning and research, which are essential for the development and implementation of kelp forest management aimed at ensuring the short-term and long-term survival of intertidal kelp and their ecosystem services along the Pacific coasts of South America.

## Supporting information

S1 Table List of additional papers reviewed to obtain data on the occurrence of *L. nigrescens*, *L. berteroana* and *L. spicata.* (DOCX)

S2 Table Database consulted on GBIF (“GBIF”; https://www.gbif.org/). Google Scholar (only for public project of Chile and Peru) and on ISI-Web of science).
(DOCX)

S3 Table Ranges of current and projected environmental variables used to the distribution models of *Lessonia berteroana* and *Lessonia spicata* for RCP2.6, RCP4.5, RCP6.0, and RCP8.5. Sea surface temperature (°C) maximum, mean, and minimum; Salinity (PSS), maximum, mean, and minimum; and velocity (ms^-1^) maximum and mean.
(DOCX)

S4 Fig Response curves (mean ± 1 SD) from 50 MaxEnt replicates for each environmental variable in *L. berteroana*; ranges with mean suitability > 0.5 highlighted. a) Minimum surface temperature (~11.5 to ~13.9 °C); b) Mean surface temperature (~14.8 to ~17.5 °C); c) Maximum surface temperature (~18.8 to ~22.2 °C); d) Minimum surface salinity (~34.5 PSS); e) Mean surface salinity (~34.6 PSS); f) Maximum surface salinity (~34.8 to ~35.0 PSS); g) Mean surface velocity (~0.21 to ~0.46 ms^-1^); h) Maximum surface velocity (~0.42 to ~0.97 ms^-1^). Temperature response curves (a-c; minimum, mean, maximum) and salinity response curves (d-f; minimum, mean, maximum) exhibit unimodal patterns, whereas velocity response curves (g-h; mean and maximum) display an asymptotic form.
(TIF)

S5 Fig Response curves (mean ± 1 SD) from 50 MaxEnt replicates for each environmental variable in *L. spicata*; ranges with mean suitability > 0.5 highlighted. a) Minimum surface temperature (~10.0 to ~12.0 °C); b) Mean surface temperature (~12.5 to ~14.8 °C); c) Maximum surface temperature (~15.0 to ~29.0 °C); d) Minimum surface salinity (~22.5 to ~33.5 PSS); e) Mean surface salinity (~28.0 to ~33.6 PSS and ~34.5 PSS); f) Maximum surface salinity (~34.5 PSS); g) Mean surface velocity (~0.05 to ~0.1 ms^-1^ and ~0.19 to ~0.33 ms^-1^); h) Maximum surface velocity (~0.1 to ~0.2 ms^-1^ and ~0.39 to ~0.7 ms^-1^). Temperature response curves (a-c; minimum, mean, maximum) and maximum salinity response curves (e) exhibit unimodal patterns, whereas minimum salinity (c), mean salinity (d) and velocity response curves (g-h; mean and maximum) display bimodal patterns responses.
(TIF)
