# Supplementary material for: Kelps on the move: Potential future distribution areas in the face of climate change, on the Pacific coast of South America
Source: PLoS One. 2025 Sep 23;20(9):e0332591. doi: 10.1371/journal.pone.0332591 (PMC12456798; doi:10.1371/journal.pone.0332591)
Supplement: S1 Table — (DOCX) [file pone.0332591.s001.docx]

**S1 Table. List of additional papers reviewed to obtain data on the occurrence of *L. nigrescens*, *L. berteroana* and *L. spicata*.**

Aguilera MA, Broitman BR, Thiel M. Spatial variability in community composition on a granite breakwater versus natural rocky shores: lack of microhabitats suppresses intertidal biodiversity. Mar Pollut Bull. 2014; 87(1-2): 257-268.

Aguilera MA, Valdivia N, Broitman BR. Herbivore-alga interaction strength influences spatial heterogeneity in a kelp-dominated intertidal community. PloS one. 2015.10(9): e0137287.

Álvarez-Campos P, Fernández-Leborans G, Verdes A, San Martín G, Martin D, Riesgo A. The tag-along friendship: epibiotic protozoans and syllid polychaetes. Implications for the taxonomy of Syllidae (Annelida), and description of three new species of Rhabdostyla and Cothurnia (Ciliophora, Peritrichia). Zool J Linn Soc. 2014. 172(2): 265-281.

Beratto-Ramos A, Castillo-Felices RDP, Troncoso-Leon NA, Agurto-Muñoz A, Agurto-Muñoz C. Selection criteria for high-value biomass: seasonal and morphological variation of polyphenolic content and antioxidant capacity in two brown macroalgae. J Appl Phycol. 2019. 31(1): 653-664.

Casares FA, Faugeron S. Higher reproductive success for chimeras than solitary individuals in the kelp Lessonia spicata but no benefit for individual genotypes. Evol Ecol. 2016. 30(5): 953-972.

Cruces E, Rojas-Lillo Y, Ramirez-Kushel E, Atala E, López-Alarcón C, Lissi E, Gómez I. Comparison of different techniques for the preservation and extraction of phlorotannins in the kelp *Lessonia spicata* (Phaeophyceae): assays of DPPH, ORAC-PGR, and ORAC-FL as testing methods. J Appl Phycol.2016. 28(1): 573-580.

Espinoza-González C, Meynard A, Núñez A, Castañeda F, Oyarzo-Miranda C, Latorre-Padilla N, Contreras-Porcia L. Assessment of the independent and combined effects of copper and polycyclic aromatic hydrocarbons on gametogenesis and sporophyte development of the kelp *Lessonia spicata* (Phaeophyceae, Ochrophyta). J Appl Phycol. 2021. 1-12.

Flores-Molina MR, Thomas D, Lovazzano C, Núñez A, Zapata J, Kumar M, Contreras-Porcia, L. Desiccation stress in intertidal seaweeds: Effects on morphology, antioxidant responses and photosynthetic performance. Aquat Bot. 2014. 113: 90-99.

Goecke F, Aránguiz-Acuña A, Palacios M, Muñoz-Muga P, Rucki M, Vítová M. Latitudinal distribution of lanthanides contained in macroalgae in Chile: An inductively coupled plasma-mass spectrometric (ICP-MS) determination. J Appl Phycol. 2017. 29(4): 2117-2128.

Gómez I, Español S, Véliz K, Huovinen P. Spatial distribution of phlorotannins and its relationship with photosynthetic UV tolerance and allocation of storage carbohydrates in blades of the kelp *Lessonia spicata*. Mar Biol. 2016. 163(5): 110.

González AV, Santelices B. Frequency of chimerism in populations of the kelp *Lessonia spicata* in central Chile. PloS one, 1.2017. 2(2): e0169182.

González AV, Borras-Chavez R, Beltrán J, Flores V, Vásquez JA, Santelices B. Morphological, ultrastructural, and genetic characterization of coalescence in the intertidal and shallow subtidal kelps *Lessonia spicata* and *L. berteroana* (Laminariales, Heterokontophyta). J Appl Phycol. 2014. 26(2):1107-1113.

González AV, Beltrán J, Hiriart‐Bertrand L, Flores V, de Reviers B, Correa JA, Santelices B. Identification of cryptic species in the *Lessonia nigrescens* complex (Phaeophyceae, Laminariales). J Phycol. 2012. 48(5): 1153-1165.

Guajardo E, Correa JA, Contreras-Porcia L. Role of abscisic acid (ABA) in activating antioxidant tolerance responses to desiccation stress in intertidal seaweed species. Planta. 2016. 243(3): 767-781.

Gutow L, Poore AG, Díaz Poblete MA, Villalobos V, Thiel M. Small burrowing amphipods cause major damage in a large kelp. Proc R Soc B. 2020. 287. 20200330. 10.1098/rspb.2020.0330.

Herrera I, Bustamante R. Foto de la portada: *Schottera nicaeensis* (por: Erasmo Macaya). Boletín de la Red Latinoamericana para el Estudio de Especies Invasoras. 2014. 4(1).

Koch K, Thiel M, Tellier F, Hagen W, Graeve M, Tala F. el al. Species separation within the *Lessonia nigrescens* complex (Phaeophyceae, Laminariales) is mirrored by ecophysiological traits. Bot Mar. 2015. 58(2): 81-92.

López-Cristoffanini C, Tellier F, Otaíza R, Correa JA, Contreras-Porcia L. Tolerance to air exposure: a feature driving the latitudinal distribution of two sibling kelp species. Bot Mar. 2013. 56(5-6): 431-440.

López P, Jaramillo E. Uso de organismos intermareales del litoral rocoso como bio-indicadores de deformación continental cosísmica en el centro sur de Chile. RMBO. 2018. 53(1): 61-73.

Lozano-Muñoz I, Castellano G, Bueno G, Wacyk J. Herbivorous fish (*Medialuna ancietae*) as a sustainable alternative for nutrition security in Northern Chile. Sci Rep. 2022. 12, 1619. https://doi.org/10.1038/s41598-021-04628-3

Murrie MAC. Análisis de la evolución temporal de la variabilidad genética en poblaciones de *Lessonia spicata* después del terremoto 27/F. 2015. (Doctoral dissertation, Universidad Austral de Chile).

Murúa P, Edrada-Ebel R, Muñoz L, Soldatou S, Legrave N, Müller DG et al.. Morphological, genotypic and metabolomic signatures confirm interfamilial hybridization between the ubiquitous kelps *Macrocystis* (Arthrothamnaceae) and *Lessonia* (Lessoniaceae). Sci Rep.2020. 10(1): 1-15.

Murúa P, Patiño DJ, Leiva FP, Muñoz L, Müller DG, Küpper FC et al. Gall disease in the alginophyte *Lessonia berteroana*: A pathogenic interaction linked with host adulthood in a seasonal-dependant manner. Algal Res.2019. 39: 101435.

Olate-Gallegos C, Barriga A, Vergara, C., Fredes, C., García, P., Giménez, B., & Robert, P. (2019). Identification of polyphenols from Chilean brown seaweeds extracts by LC-DAD-ESI-MS/MS. J Aquat Food Prod Technol. 2019. 28(4): 375-391.

Ortega KJ, Sáez CA, Macaya EC. Changes in invertebrate assemblages inhabiting *Lessonia spicata* (Phaeophyceae) holdfasts after the 2010 earthquake-mediated coastal uplift in Chile. RMBO. 2014. 49(1): 129-134.

Oyarzo-Miranda C, Latorre N, Meynard A, Rivas J, Bulboa C, Contreras-Porcia L. Coastal pollution from the industrial park Quintero Bay of central Chile: Effects on abundance, morphology, and development of the kelp *Lessonia spicata* (Phaeophyceae). PloS one. 2020.15(10): e0240581.

Parada GM, Martínez EA, Aguilera MA, Oróstica MH, Broitman BR. Interactions between kelp spores and encrusting and articulated corallines: recruitment challenges for *Lessonia spicata*. Bot Mar. 2017. 60(6): 619-625.

Parada GM, Tellier F, Martínez EA. Spore dispersal in the intertidal kelp *Lessonia spicata*: Macrochallenges for the harvested *Lessonia* species complex at microscales of space and time. Bot Mar. 2016. 59(4): 283-289.

Poore AG, Gutow L, Pantoja JF, Tala F, Madariaga DJ, Thiel M. Major consequences of minor damage: impacts of small grazers on fast-growing kelps. Oecologia. 2014. 174(3): 789-801.

Quintanilla-Ahumada D, Quijón PA, Navarro JM, Pulgar J, Duarte C. Living on a trophic subsidy: Algal quality drives an upper-shore herbivore’s consumption, preference and absorption but not growth rates. Plos one. 2018. 13(4): e0196121.

Ritter A, Cabioch L, Brillet-Guéguen L, Corre E, Cosse A, Dartevelle L. et al. Herbivore-induced chemical and molecular responses of the kelps Laminaria digitata and Lessonia spicata. PLoS One, 2017. 12(3): e0173315.

Rodríguez D, Oróstica MH, Vásquez JA. Coalescence in wild organisms of the intertidal population of *Lessonia berteroana* in northern Chile: management and sustainability effects. J Appl Phycol. 2014.26(2): 1115-1122.

Tellier F, Meynard AP, Correa JA, Faugeron S, Valero M. Phylogeographic analyses of the 30° S south-east Pacific biogeographic transition zone establish the occurrence of a sharp genetic discontinuity in the kelp *Lessonia nigrescens*: Vicariance or parapatry? Mol Phylogenet Evol. 2009. 53(3): 679-693.

Troncoso N, Saavedra R, Olivares A, Farías J, San-Martín S, Urrutia H, Agurto C. Identificación de compuestos antibacterianos en macroalgas presentes en la Región del Biobío, Chile. RBMO. 2015. 50: 199-204.

Vásquez J. Evaluación de biomasa y análisis del estado de explotación de las praderas naturales de algas pardas (huiro negro, huiro palo y huiro flotador) en las áreas de libre acceso de la XV región de Arica y Parinacota, I región de Tarapacá y II región de Antofagasta. Universidad Católica del Norte, proyecto FIP 2017-52. 2018

Vega JA, Broitman BR, Vásquez JA. Monitoring the sustainability of *Lessonia nigrescens* (Laminariales, Phaeophyceae) in northern Chile under strong harvest pressure. J Appl Phycol. 2014. 26(2): 791-801.

Vega JM. Fauna asociada a discos de adhesión del complejo *Lessonia nigrescens*: ¿Es un indicador de integridad ecológica en praderas explotadas de huiro negro, en el norte de Chile? Lat Am J Aquat Res. 2016. 44(3): 623-637.

Vega JM, Toledo PH. The chemical composition of *Lessonia berteroana* (ex *L. nigrescens*) in kelp harvest management and open access areas near Coquimbo, Chile. Lat Am J Aquat Res. 2018. 46(2): 258-267.

Vega JM, Asorey CM, Piaget N. Asociación *Scurria-Lessonia*, indicador de integridad ecológica en praderas explotadas de huiro negro *Lessonia berteroana* (ex *L. nigrescens*) en el norte de Chile. RBMO. 2016. 51(2): 337-345.

Vega JM, Valdebenito M, Caillaux L, Bravo J. Abundancia y estructura poblacional de dos recursos pesqueros bentónicos fuera y dentro del área de una concesión marítima portuaria en Caldera, Región de Atacama, Chile. RBMO. 2019. 54(2): 232-237.

Velásquez C, Jaramillo E, Camus PA, Manzano M, Sánchez R. Biota del intermareal rocoso expuesto de la Isla Grande de Chiloé, Archipiélago de Chiloé, Chile: Patrones de diversidad e implicancias ecológicas y biogeográficas. RBMO. 2016. 51(1): 33-50.

Westermeier R, Murúa P, Patiño DJ, Manoli G, Müller DG. Evaluation of kelp harvest strategies: recovery of *Lessonia berteroana* (Phaeophyceae, Laminariales) in Pan de Azucar, Atacama, Chile. J Appl Phycol. 2019. 31(1): 575-585.

Zúñiga A, Sáez CA, Trabal A, Figueroa FL, Pardo D, Navarrete C, Rodríguez-Rojas F, Moenne F, Celis-Plá, PS. Seasonal Photoacclimation and Vulnerability Patterns in the Brown Macroalga *Lessonia spicata* (Ochrophyta). Water. 2021.13(1): 6.

Pérez-Araneda K, Zevallos S, Arakaki N, Gamarra A, Carbajal P, Tellier F. *Lessonia berteroana* en Perú: Comprobación de la identidad de la especie y diversidad genética en el borde norte de distribución. RBMO. 2020. 55(3): 270-276.
