# Supplementary material for: Kelps on the move: Potential future distribution areas in the face of climate change, on the Pacific coast of South America
Source: PLoS One. 2025 Sep 23;20(9):e0332591. doi: 10.1371/journal.pone.0332591 (PMC12456798; doi:10.1371/journal.pone.0332591)
Supplement: S2 Table — Google Scholar (only for public project of Chile and Peru) and on ISI-Web of science). (DOCX) [file pone.0332591.s002.docx]

**S2 Table. Database consulted on GBIF (“GBIF”; https://www.gbif.org/). Google Scholar (only for public project of Chile and Peru) and on ISI-Web of science).**

|  | **Specie** | **Latitude** | **Longitude** |
| --- | --- | --- | --- |
| 1 | *Lessonia berteroana* | -28.4879 | -71.2532 |
| 2 | *Lessonia berteroana* | -20.2232 | -70.1668 |
| 3 | *Lessonia berteroana* | -26.1490 | -70.6909 |
| 4 | *Lessonia berteroana* | -20.8142 | -70.2009 |
| 5 | *Lessonia berteroana* | -20.4311 | -70.1667 |
| 6 | *Lessonia berteroana* | -20.7040 | -70.1908 |
| 7 | *Lessonia berteroana* | -16.8080 | -72.3334 |
| 8 | *Lessonia berteroana* | -21.1100 | -70.1300 |
| 9 | *Lessonia berteroana* | -17.1023 | -71.9048 |
| 10 | *Lessonia berteroana* | -27.0635 | -70.8335 |
| 11 | *Lessonia berteroana* | -27.0551 | -70.8334 |
| 12 | *Lessonia berteroana* | -23.6517 | -70.4168 |
| 13 | *Lessonia berteroana* | -26.3214 | -70.6671 |
| 14 | *Lessonia berteroana* | -26.2251 | -70.6669 |
| 15 | *Lessonia berteroana* | -26.2964 | -70.6667 |
| 16 | *Lessonia berteroana* | -26.2588 | -70.6668 |
| 17 | *Lessonia berteroana* | -26.1298 | -70.6667 |
| 18 | *Lessonia berteroana* | -26.3796 | -70.6715 |
| 19 | *Lessonia berteroana* | -26.1931 | -70.6726 |
| 20 | *Lessonia berteroana* | -26.1587 | -70.6726 |
| 21 | *Lessonia berteroana* | -26.5598 | -70.6832 |
| 22 | *Lessonia berteroana* | -26.4634 | -70.6906 |
| 23 | *Lessonia berteroana* | -26.4291 | -70.6907 |
| 24 | *Lessonia berteroana* | -26.3919 | -70.6975 |
| 25 | *Lessonia berteroana* | -26.5293 | -70.6996 |
| 26 | *Lessonia berteroana* | -26.5661 | -70.7006 |
| 27 | *Lessonia berteroana* | -26.4976 | -70.7037 |
| 28 | *Lessonia berteroana* | -26.6119 | -70.7502 |
| 29 | *Lessonia berteroana* | -26.6629 | -70.7503 |
| 30 | *Lessonia berteroana* | -26.7350 | -70.7501 |
| 31 | *Lessonia berteroana* | -26.7520 | -70.7500 |
| 32 | *Lessonia berteroana* | -26.6856 | -70.7501 |
| 33 | *Lessonia berteroana* | -26.7641 | -70.7779 |
| 34 | *Lessonia berteroana* | -26.9785 | -70.7896 |
| 35 | *Lessonia berteroana* | -26.7997 | -70.7938 |
| 36 | *Lessonia berteroana* | -27.0115 | -70.8338 |
| 37 | *Lessonia berteroana* | -26.9454 | -70.8044 |
| 38 | *Lessonia berteroana* | -26.8344 | -70.8335 |
| 39 | *Lessonia berteroana* | -27.0357 | -70.8335 |
| 40 | *Lessonia berteroana* | -26.8908 | -70.8335 |
| 41 | *Lessonia berteroana* | -26.8651 | -70.8334 |
| 42 | *Lessonia berteroana* | -27.0503 | -70.8334 |
| 43 | *Lessonia berteroana* | -27.0698 | -70.8424 |
| 44 | *Lessonia berteroana* | -27.0488 | -70.8475 |
| 45 | *Lessonia berteroana* | -27.0755 | -70.8648 |
| 46 | *Lessonia berteroana* | -27.0923 | -70.8663 |
| 47 | *Lessonia berteroana* | -27.1197 | -70.8683 |
| 48 | *Lessonia berteroana* | -27.4972 | -70.9168 |
| 49 | *Lessonia berteroana* | -27.4632 | -70.9169 |
| 50 | *Lessonia berteroana* | -27.5141 | -70.9169 |
| 51 | *Lessonia berteroana* | -27.6048 | -70.9169 |
| 52 | *Lessonia berteroana* | -27.5563 | -70.9168 |
| 53 | *Lessonia berteroana* | -27.1272 | -70.9035 |
| 54 | *Lessonia berteroana* | -27.6308 | -70.9221 |
| 55 | *Lessonia berteroana* | -27.4187 | -70.9262 |
| 56 | *Lessonia berteroana* | -27.3133 | -70.9327 |
| 57 | *Lessonia berteroana* | -27.0985 | -70.9312 |
| 58 | *Lessonia berteroana* | -27.2777 | -70.9379 |
| 59 | *Lessonia berteroana* | -27.3841 | -70.9404 |
| 60 | *Lessonia berteroana* | -27.6567 | -70.9499 |
| 61 | *Lessonia berteroana* | -27.2509 | -70.9529 |
| 62 | *Lessonia berteroana* | -27.2221 | -71.0004 |
| 63 | *Lessonia berteroana* | -27.1277 | -70.9543 |
| 64 | *Lessonia berteroana* | -27.1925 | -71.0003 |
| 65 | *Lessonia berteroana* | -27.3575 | -70.9657 |
| 66 | *Lessonia berteroana* | -27.1580 | -70.9729 |
| 67 | *Lessonia berteroana* | -27.6606 | -70.9822 |
| 68 | *Lessonia berteroana* | -27.6672 | -71.0149 |
| 69 | *Lessonia berteroana* | -27.6507 | -71.0367 |
| 70 | *Lessonia berteroana* | -27.7111 | -71.0421 |
| 71 | *Lessonia berteroana* | -27.7383 | -71.0532 |
| 72 | *Lessonia berteroana* | -27.7655 | -71.0834 |
| 73 | *Lessonia berteroana* | -27.8173 | -71.0850 |
| 74 | *Lessonia berteroana* | -27.8773 | -71.1053 |
| 75 | *Lessonia berteroana* | -27.8444 | -71.1116 |
| 76 | *Lessonia berteroana* | -27.8972 | -71.1178 |
| 77 | *Lessonia berteroana* | -27.9619 | -71.1436 |
| 78 | *Lessonia berteroana* | -27.9968 | -71.1448 |
| 79 | *Lessonia berteroana* | -22.5500 | -70.2667 |
| 80 | *Lessonia berteroana* | -29.2333 | -71.5004 |
| 81 | *Lessonia berteroana* | -29.9167 | -71.3500 |
| 82 | *Lessonia berteroana* | -28.1833 | -71.1669 |
| 83 | *Lessonia berteroana* | -29.2000 | -71.5002 |
| 84 | *Lessonia berteroana* | -29.8167 | -71.3338 |
| 85 | *Lessonia berteroana* | -29.1806 | -71.5002 |
| 86 | *Lessonia berteroana* | -19.1833 | -70.2667 |
| 87 | *Lessonia berteroana* | -19.6000 | -70.2503 |
| 88 | *Lessonia berteroana* | -20.8000 | -70.2000 |
| 89 | *Lessonia berteroana* | -23.7667 | -70.5002 |
| 90 | *Lessonia berteroana* | -26.1500 | -70.6667 |
| 91 | *Lessonia berteroana* | -27.2000 | -71.0005 |
| 92 | *Lessonia berteroana* | -27.6833 | -71.0333 |
| 93 | *Lessonia berteroana* | -28.0667 | -71.1672 |
| 94 | *Lessonia berteroana* | -28.4500 | -71.2167 |
| 95 | *Lessonia berteroana* | -16.9663 | -72.1670 |
| 96 | *Lessonia berteroana* | -17.6166 | -71.3443 |
| 97 | *Lessonia berteroana* | -27.2480 | -71.0003 |
| 98 | *Lessonia berteroana* | -30.0833 | -71.4170 |
| 99 | *Lessonia berteroana* | -27.7570 | -71.0836 |
| 100 | *Lessonia berteroana* | -28.2500 | -71.1667 |
| 101 | *Lessonia berteroana* | -30.1030 | -71.4170 |
| 102 | *Lessonia berteroana* | -30.8640 | -71.6830 |
| 103 | *Lessonia berteroana* | -29.2590 | -71.5350 |
| 104 | *Lessonia berteroana* | -30.8333 | -71.6833 |
| 105 | *Lessonia berteroana* | -30.0167 | -71.4169 |
| 106 | *Lessonia berteroana* | -27.0520 | -70.8421 |
| 107 | *Lessonia berteroana* | -27.0508 | -70.8496 |
| 108 | *Lessonia berteroana* | -28.1667 | -71.1667 |
| 109 | *Lessonia berteroana* | -20.2167 | -70.1668 |
| 110 | *Lessonia berteroana* | -19.2119 | -70.2764 |
| 111 | *Lessonia berteroana* | -19.5981 | -70.2506 |
| 112 | *Lessonia berteroana* | -19.5744 | -70.2047 |
| 113 | *Lessonia berteroana* | -20.4739 | -70.1689 |
| 114 | *Lessonia berteroana* | -20.6717 | -70.1878 |
| 115 | *Lessonia berteroana* | -21.0356 | -70.1667 |
| 116 | *Lessonia berteroana* | -21.7330 | -70.1668 |
| 117 | *Lessonia berteroana* | -23.3586 | -70.6050 |
| 118 | *Lessonia berteroana* | -25.6305 | -70.6669 |
| 119 | *Lessonia berteroana* | -25.6639 | -70.6775 |
| 120 | *Lessonia berteroana* | -19.2444 | -70.2808 |
| 121 | *Lessonia berteroana* | -19.5803 | -70.2064 |
| 122 | *Lessonia berteroana* | -19.5981 | -70.2504 |
| 123 | *Lessonia berteroana* | -20.6717 | -70.1878 |
| 124 | *Lessonia berteroana* | -21.0356 | -70.1667 |
| 125 | *Lessonia berteroana* | -21.7336 | -70.1668 |
| 126 | *Lessonia berteroana* | -22.3933 | -70.2500 |
| 127 | *Lessonia berteroana* | -24.8327 | -70.5837 |
| 128 | *Lessonia berteroana* | -25.6318 | -70.6670 |
| 129 | *Lessonia berteroana* | -25.6633 | -70.6781 |
| 130 | *Lessonia berteroana* | -19.5883 | -70.2502 |
| 131 | *Lessonia berteroana* | -19.5936 | -70.2504 |
| 132 | *Lessonia berteroana* | -20.8867 | -70.1671 |
| 133 | *Lessonia berteroana* | -15.4000 | -75.1668 |
| 134 | *Lessonia berteroana* | -16.2167 | -73.6667 |
| 135 | *Lessonia berteroana* | -17.6500 | -71.3500 |
| 136 | *Lessonia berteroana* | -30.1000 | -71.4171 |
| 137 | *Lessonia spicata* | -32.2394 | -71.5244 |
| 138 | *Lessonia spicata* | -36.5996 | -72.9776 |
| 139 | *Lessonia spicata* | -31.9647 | -71.5014 |
| 140 | *Lessonia spicata* | -36.5910 | -72.9796 |
| 141 | *Lessonia spicata* | -36.5947 | -72.9783 |
| 142 | *Lessonia spicata* | -33.3136 | -71.6668 |
| 143 | *Lessonia spicata* | -36.5946 | -72.9788 |
| 144 | *Lessonia spicata* | -36.1386 | -72.8337 |
| 145 | *Lessonia spicata* | -36.5948 | -72.9771 |
| 146 | *Lessonia spicata* | -36.5993 | -72.9776 |
| 147 | *Lessonia spicata* | -37.1481 | -73.5861 |
| 148 | *Lessonia spicata* | -31.7294 | -71.5464 |
| 149 | *Lessonia spicata* | -39.7963 | -73.4171 |
| 150 | *Lessonia spicata* | -36.5942 | -72.9787 |
| 151 | *Lessonia spicata* | -39.7203 | -73.4058 |
| 152 | *Lessonia spicata* | -32.9567 | -71.5507 |
| 153 | *Lessonia spicata* | -33.5019 | -71.6327 |
| 154 | *Lessonia spicata* | -33.5017 | -71.6322 |
| 155 | *Lessonia spicata* | -36.5941 | -72.9788 |
| 156 | *Lessonia spicata* | -36.5951 | -72.9771 |
| 157 | *Lessonia spicata* | -36.5953 | -72.9781 |
| 158 | *Lessonia spicata* | -36.5938 | -72.9788 |
| 159 | *Lessonia spicata* | -36.8110 | -73.1736 |
| 160 | *Lessonia spicata* | -36.5946 | -72.9788 |
| 161 | *Lessonia spicata* | -36.5935 | -72.9789 |
| 162 | *Lessonia spicata* | -36.7724 | -73.2120 |
| 163 | *Lessonia spicata* | -36.5947 | -72.9786 |
| 164 | *Lessonia spicata* | -36.5948 | -72.9789 |
| 165 | *Lessonia spicata* | -36.5937 | -72.9787 |
| 166 | *Lessonia spicata* | -36.5953 | -72.9780 |
| 167 | *Lessonia spicata* | -36.5946 | -72.9779 |
| 168 | *Lessonia spicata* | -36.5954 | -72.9781 |
| 169 | *Lessonia spicata* | -36.5940 | -72.9789 |
| 170 | *Lessonia spicata* | -36.5931 | -72.9785 |
| 171 | *Lessonia spicata* | -36.5960 | -72.9779 |
| 172 | *Lessonia spicata* | -34.6406 | -72.0462 |
| 173 | *Lessonia spicata* | -32.9738 | -71.5464 |
| 174 | *Lessonia spicata* | -36.5958 | -72.9785 |
| 175 | *Lessonia spicata* | -36.8080 | -73.1743 |
| 176 | *Lessonia spicata* | -36.7636 | -73.2043 |
| 177 | *Lessonia spicata* | -37.0070 | -73.1855 |
| 178 | *Lessonia spicata* | -37.6870 | -73.6668 |
| 179 | *Lessonia spicata* | -41.9471 | -74.0837 |
| 180 | *Lessonia spicata* | -36.5937 | -72.9790 |
| 181 | *Lessonia spicata* | -36.5998 | -72.9781 |
| 182 | *Lessonia spicata* | -36.5931 | -72.9791 |
| 183 | *Lessonia spicata* | -36.5941 | -72.9780 |
| 184 | *Lessonia spicata* | -36.5949 | -72.9787 |
| 185 | *Lessonia spicata* | -36.5957 | -72.9777 |
| 186 | *Lessonia spicata* | -36.5941 | -72.9787 |
| 187 | *Lessonia spicata* | -36.5938 | -72.9789 |
| 188 | *Lessonia spicata* | -36.5930 | -72.9785 |
| 189 | *Lessonia spicata* | -36.5958 | -72.9776 |
| 190 | *Lessonia spicata* | -36.5979 | -72.9772 |
| 191 | *Lessonia spicata* | -36.5250 | -72.9540 |
| 192 | *Lessonia spicata* | -36.5955 | -72.9776 |
| 193 | *Lessonia spicata* | -36.5962 | -72.9775 |
| 194 | *Lessonia spicata* | -36.5944 | -72.9787 |
| 195 | *Lessonia spicata* | -36.5241 | -72.9531 |
| 196 | *Lessonia spicata* | -36.5929 | -72.9788 |
| 197 | *Lessonia spicata* | -36.7728 | -73.2131 |
| 198 | *Lessonia spicata* | -37.5787 | -73.6440 |
| 199 | *Lessonia spicata* | -36.5943 | -72.9790 |
| 200 | *Lessonia spicata* | -36.8091 | -73.1734 |
| 201 | *Lessonia spicata* | -32.7406 | -71.4995 |
| 202 | *Lessonia spicata* | -36.5450 | -72.9369 |
| 203 | *Lessonia spicata* | -37.5943 | -73.6696 |
| 204 | *Lessonia spicata* | -36.5875 | -72.9833 |
| 205 | *Lessonia spicata* | -36.5952 | -72.9781 |
| 206 | *Lessonia spicata* | -36.6044 | -72.9789 |
| 207 | *Lessonia spicata* | -36.5536 | -72.9767 |
| 208 | *Lessonia spicata* | -36.5969 | -72.9767 |
| 209 | *Lessonia spicata* | -36.6042 | -72.9785 |
| 210 | *Lessonia spicata* | -36.5937 | -72.9791 |
| 211 | *Lessonia spicata* | -40.5600 | -73.7504 |
| 212 | *Lessonia spicata* | -32.5549 | -71.4547 |
| 213 | *Lessonia spicata* | -33.0470 | -71.6080 |
| 214 | *Lessonia spicata* | -33.0470 | -71.6080 |
| 215 | *Lessonia spicata* | -33.0448 | -71.6743 |
| 216 | *Lessonia spicata* | -33.0470 | -71.6080 |
| 217 | *Lessonia spicata* | -33.0470 | -71.6080 |
| 218 | *Lessonia spicata* | -33.0470 | -71.6080 |
| 219 | *Lessonia spicata* | -32.5554 | -71.4575 |
| 220 | *Lessonia spicata* | -33.0470 | -71.6080 |
| 221 | *Lessonia spicata* | -33.0470 | -71.6080 |
| 222 | *Lessonia spicata* | -32.5522 | -71.4619 |
| 223 | *Lessonia spicata* | -33.5592 | -71.6256 |
| 224 | *Lessonia spicata* | -33.0313 | -71.6154 |
| 225 | *Lessonia spicata* | -33.0265 | -71.5835 |
| 226 | *Lessonia spicata* | -32.9694 | -71.5458 |
| 227 | *Lessonia spicata* | -30.2928 | -71.6087 |
| 228 | *Lessonia spicata* | -31.8955 | -71.5038 |
| 229 | *Lessonia spicata* | -31.8732 | -71.5032 |
| 230 | *Lessonia spicata* | -32.1170 | -71.5839 |
| 231 | *Lessonia spicata* | -31.8514 | -71.5081 |
| 232 | *Lessonia spicata* | -31.7515 | -71.5077 |
| 233 | *Lessonia spicata* | -32.0594 | -71.5249 |
| 234 | *Lessonia spicata* | -31.7922 | -71.5178 |
| 235 | *Lessonia spicata* | -32.1005 | -71.5838 |
| 236 | *Lessonia spicata* | -31.8372 | -71.5221 |
| 237 | *Lessonia spicata* | -31.7021 | -71.5440 |
| 238 | *Lessonia spicata* | -31.6787 | -71.5455 |
| 239 | *Lessonia spicata* | -31.6189 | -71.5836 |
| 240 | *Lessonia spicata* | -31.5505 | -71.5698 |
| 241 | *Lessonia spicata* | -31.5247 | -71.5702 |
| 242 | *Lessonia spicata* | -31.5975 | -71.5835 |
| 243 | *Lessonia spicata* | -31.5754 | -71.5778 |
| 244 | *Lessonia spicata* | -31.4767 | -71.5835 |
| 245 | *Lessonia spicata* | -31.4594 | -71.5848 |
| 246 | *Lessonia spicata* | -31.3161 | -71.6672 |
| 247 | *Lessonia spicata* | -31.3421 | -71.6190 |
| 248 | *Lessonia spicata* | -31.3672 | -71.6198 |
| 249 | *Lessonia spicata* | -31.2973 | -71.6669 |
| 250 | *Lessonia spicata* | -31.2346 | -71.6669 |
| 251 | *Lessonia spicata* | -31.2113 | -71.6670 |
| 252 | *Lessonia spicata* | -31.1136 | -71.6667 |
| 253 | *Lessonia spicata* | -32.7419 | -71.4925 |
| 254 | *Lessonia spicata* | -32.7092 | -71.4886 |
| 255 | *Lessonia spicata* | -32.5844 | -71.4561 |
| 256 | *Lessonia spicata* | -32.3333 | -71.5005 |
| 257 | *Lessonia spicata* | -32.6333 | -71.4333 |
| 258 | *Lessonia spicata* | -32.9553 | -71.5478 |
| 259 | *Lessonia spicata* | -39.8500 | -73.3833 |
| 260 | *Lessonia spicata* | -32.6500 | -71.4833 |
| 261 | *Lessonia spicata* | -33.9333 | -71.8833 |
| 262 | *Lessonia spicata* | -38.7167 | -73.4170 |
| 263 | *Lessonia spicata* | -33.5025 | -71.6331 |
| 264 | *Lessonia spicata* | -39.7667 | -73.4167 |
| 265 | *Lessonia spicata* | -29.2167 | -71.5331 |
| 266 | *Lessonia spicata* | -30.7333 | -71.7505 |
| 267 | *Lessonia spicata* | -33.4547 | -71.6731 |
| 268 | *Lessonia spicata* | -36.8069 | -73.1772 |
| 269 | *Lessonia spicata* | -41.8667 | -74.0167 |
| 270 | *Lessonia spicata* | -33.4832 | -71.6668 |
| 271 | *Lessonia spicata* | -29.0667 | -71.5002 |
| 272 | *Lessonia spicata* | -29.1500 | -71.5002 |
| 273 | *Lessonia spicata* | -30.8333 | -71.6833 |
| 274 | *Lessonia spicata* | -32.3167 | -71.5002 |
| 275 | *Lessonia spicata* | -33.4586 | -71.6667 |
| 276 | *Lessonia spicata* | -29.1681 | -71.5002 |
| 277 | *Lessonia spicata* | -32.6500 | -71.4333 |
| 278 | *Lessonia spicata* | -30.9311 | -71.6706 |
| 279 | *Lessonia spicata* | -32.6536 | -71.4436 |
| 280 | *Lessonia spicata* | -33.4394 | -71.6883 |
| 281 | *Lessonia spicata* | -33.1833 | -71.6833 |
| 282 | *Lessonia spicata* | -36.5992 | -72.9775 |
| 283 | *Lessonia spicata* | -36.8083 | -73.1744 |
| 284 | *Lessonia spicata* | -37.1475 | -73.5808 |
| 285 | *Lessonia spicata* | -37.5778 | -73.6422 |
| 286 | *Lessonia spicata* | -38.3411 | -73.5067 |
| 287 | *Lessonia spicata* | -39.4242 | -73.2502 |
| 288 | *Lessonia spicata* | -30.7500 | -71.7502 |
| 289 | *Lessonia spicata* | -30.9167 | -71.6667 |
| 290 | *Lessonia spicata* | -31.4167 | -71.6000 |
| 291 | *Lessonia spicata* | -31.6333 | -71.5836 |
| 292 | *Lessonia spicata* | -32.6667 | -71.4500 |
| 293 | *Lessonia spicata* | -32.2333 | -71.5167 |
| 294 | *Lessonia spicata* | -32.9500 | -71.5333 |
| 295 | *Lessonia spicata* | -33.0833 | -71.7167 |
| 296 | *Lessonia spicata* | -33.3833 | -71.7000 |
| 297 | *Lessonia spicata* | -33.5000 | -71.6669 |
| 298 | *Lessonia spicata* | -33.9500 | -71.8667 |
| 299 | *Lessonia spicata* | -34.3833 | -72.0167 |
| 300 | *Lessonia spicata* | -35.3167 | -72.4167 |
| 301 | *Lessonia spicata* | -36.5000 | -72.9170 |
| 302 | *Lessonia spicata* | -40.5333 | -73.7506 |
| 303 | *Lessonia spicata* | -41.8000 | -74.0167 |
| 304 | *Lessonia spicata* | -30.8640 | -71.6830 |
| 305 | *Lessonia spicata* | -34.6894 | -72.0835 |
| 306 | *Lessonia spicata* | -34.8303 | -72.1450 |
| 307 | *Lessonia spicata* | -35.8156 | -72.5861 |
| 308 | *Lessonia spicata* | -36.5981 | -72.9753 |
| 309 | *Lessonia spicata* | -37.5800 | -73.6428 |
| 310 | *Lessonia spicata* | -36.7686 | -73.2081 |
| 311 | *Lessonia spicata* | -37.0100 | -73.1831 |
| 312 | *Lessonia spicata* | -37.6106 | -73.6669 |
| 313 | *Lessonia spicata* | -38.2422 | -73.4922 |
| 314 | *Lessonia spicata* | -38.3411 | -73.5075 |
| 315 | *Lessonia spicata* | -36.9683 | -73.5236 |
| 316 | *Lessonia spicata* | -36.9769 | -73.5342 |
| 317 | *Lessonia spicata* | -36.9861 | -73.5183 |
| 318 | *Lessonia spicata* | -37.0222 | -73.5522 |
| 319 | *Lessonia spicata* | -38.4164 | -73.8969 |
| 320 | *Lessonia spicata* | -39.8044 | -73.4171 |
| 321 | *Lessonia spicata* | -39.8169 | -73.4172 |
| 322 | *Lessonia spicata* | -40.5436 | -73.7506 |
| 323 | *Lessonia spicata* | -40.5864 | -73.7506 |
| 324 | *Lessonia spicata* | -40.6147 | -73.7533 |
| 325 | *Lessonia spicata* | -35.8333 | -72.6333 |
| 326 | *Lessonia spicata* | -36.4667 | -72.9168 |
| 327 | *Lessonia spicata* | -41.9167 | -74.0835 |
| 328 | *Lessonia spicata* | -41.9833 | -74.0838 |
| 329 | *Lessonia spicata* | -33.5000 | -71.6669 |
| 330 | *Lessonia spicata* | -32.7200 | -71.4783 |
| 331 | *Lessonia spicata* | -32.9628 | -71.5469 |
| 332 | *Lessonia spicata* | -36.7667 | -73.2000 |
| 333 | *Lessonia spicata* | -32.3456 | -71.4600 |
| 334 | *Lessonia spicata* | -32.9500 | -71.5500 |
| 335 | *Lessonia spicata* | -33.4242 | -71.7042 |
| 336 | *Lessonia spicata* | -33.5025 | -71.6336 |
|  | | | |
