# Supplementary material for: Kelps on the move: Potential future distribution areas in the face of climate change, on the Pacific coast of South America
Source: PLoS One. 2025 Sep 23;20(9):e0332591. doi: 10.1371/journal.pone.0332591 (PMC12456798; doi:10.1371/journal.pone.0332591)
Supplement: S3 Table — Sea surface temperature (°C) maximum, mean, and minimum; Salinity (PSS), maximum, mean, and minimum; and velocity (ms-1) maximum and mean. (DOCX) [file pone.0332591.s003.docx]

**S3. Table. Ranges of current and projected environmental variables used to the distribution models of *Lessonia berteroana* and *Lessonia spicata* for RCP2.6, RCP4.5, RCP6.0, and RCP8.5. Sea surface temperature (ºC) maximum, mean, and minimum; Salinity (PSS), maximum, mean, and minimum; and velocity (m s-1) maximum and mean.**

|  | **Current** | **RCP 2.6** | **RCP 4.5** | **RCP 6.0** | **RCP 8.5** |
| --- | --- | --- | --- | --- | --- |
| Maximum surface temperature | 5.70 – 27.10 | 6.05 – 27.98 | 6.40 – 29.31 | 6.22 - 28.54 | 6.26 – 23.79 |
| Mean surface temperature | 3.62 – 22.21 | 3.78 – 23.08 | 4.08 – 23.79 | 4.08 – 23.17 | 4.19 – 23.79 |
| Minimum surface temperature | 1.73 – 17.13 | 1.87 – 18.41 | 2.00 – 18.71 | 2.02 – 17.57 | 2.19 – 18.56 |
| Maximum surface salinity | 27.74 – 35.84 | 27.73 – 36.02 | 27.92 – 36.07 | 27.87 – 36.00 | 27.95 – 36.08 |
| Mean surface salinity | 25.39 – 35.40 | 25.45 – 35.50 | 25.48 - 35.51 | 25.43 - 35.47 | 25.55 - 35.54 |
| Minimum surface salinity | 23.54 – 35.09 | 23.70 – 35.11 | 23.48 - 35.23 | 23.52 - 35.19 | 24.00 - 35.23 |
| Maximum surface velocity | 0.02 – 1.00 | 0.02 – 1.00 | 0.03 – 1.01 | 0.04 – 1.00 | 0.03 – 1.01 |
| Mean surface velocity | 0.01 – 0.55 | 0.01 – 0.56 | 0.01 - 0.55 | 0.02 - 0.54 | 0.01 - 0.55 |
