# Supplementary figures and images for: Kelps on the move: Potential future distribution areas in the face of climate change, on the Pacific coast of South America
Source: PLoS One. 2025 Sep 23;20(9):e0332591. doi: 10.1371/journal.pone.0332591 (PMC12456798; doi:10.1371/journal.pone.0332591)

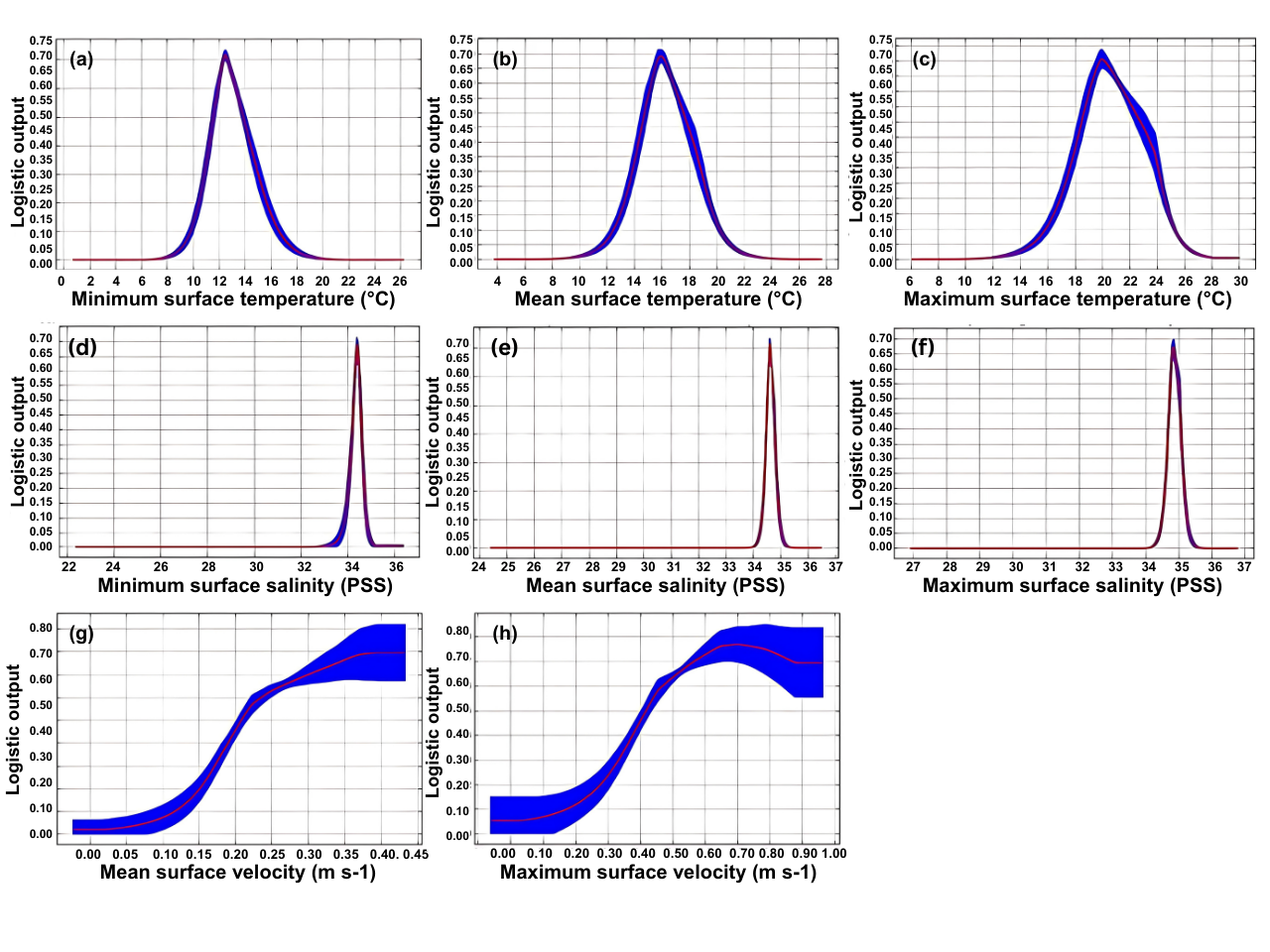

Supplement: S4 Fig — a) Minimum surface temperature (~11.5 to ~13.9 °C); b) Mean surface temperature (~14.8 to ~17.5 °C); c) Maximum surface temperature (~18.8 to ~22.2 °C); d) Minimum surface salinity (~34.5 PSS); e) Mean surface salinity (~34.6 PSS); f) Maximum surface salinity (~34.8 to ~35.0 PSS); g) Mean surface velocity (~0.21 to ~0.46 ms-1); h) Maximum surface velocity (~0.42 to ~0.97 ms-1). Temperature response curves (a-c; minimum, mean, maximum) and salinity response curves (d-f; minimum, mean, maximum) exhibit unimodal patterns, whereas velocity response curves (g-h; mean and maximum) display an asymptotic form. (TIF) [file pone.0332591.s004.tif]

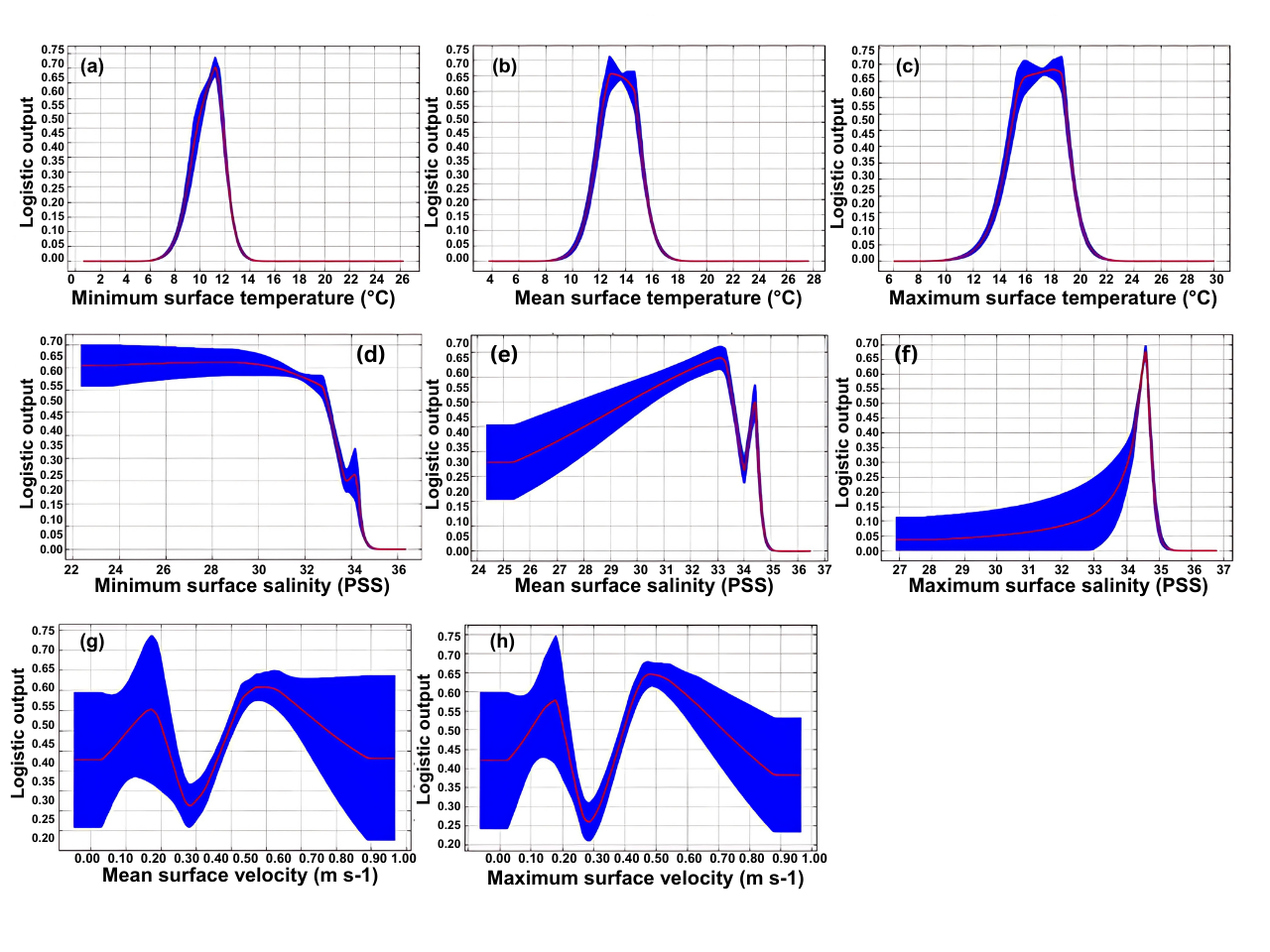

Supplement: S5 Fig — a) Minimum surface temperature (~10.0 to ~12.0 °C); b) Mean surface temperature (~12.5 to ~14.8 °C); c) Maximum surface temperature (~15.0 to ~29.0 °C); d) Minimum surface salinity (~22.5 to ~33.5 PSS); e) Mean surface salinity (~28.0 to ~33.6 PSS and ~34.5 PSS); f) Maximum surface salinity (~34.5 PSS); g) Mean surface velocity (~0.05 to ~0.1 ms-1 and ~0.19 to ~0.33 ms-1); h) Maximum surface velocity (~0.1 to ~0.2 ms-1 and ~0.39 to ~0.7 ms-1). Temperature response curves (a-c; minimum, mean, maximum) and maximum salinity response curves (e) exhibit unimodal patterns, whereas minimum salinity (c), mean salinity (d) and velocity response curves (g-h; mean and maximum) display bimodal patterns responses. (TIF) [file pone.0332591.s005.tif]
